# Veränderungen der Nierenfunktionsparameter nach Cotrimoxazoltherapie

**DOI:** 10.1007/s00108-025-01974-6

**Published:** 2025-08-23

**Authors:** John Michael Hoppe, Martin Klaus, Matthias Auer, Markus Wörnle

**Affiliations:** 1 Zentrale Notaufnahme Klinikum Innenstadt, LMU Klinikum, Ludwig-Maximilians-Universität München, Ziemssenstraße 5, 80336 München, Deutschland; 2 Medizinische Klinik und Poliklinik IV, LMU Klinikum, Ludwig-Maximilians-Universität München, München, Deutschland

**Keywords:** Akutes Nierenversagen, Cystatin C, Glomeruläre Filtrationsrate, Kreatinin, Trimethoprim/Sulfamethoxazol, Acute kidney injury, Cystatin C, Glomerular filtration rate, Creatinine, Trimethoprim/sulfamethoxazole

## Abstract

Cotrimoxazol ist ein Kombinationsantibiotikum aus den beiden Wirkstoffen Trimethoprim und Sulfamethoxazol (TMP-SMX). Es ist bekannt, dass TMP-SMX Nierenfunktionsparameter beeinflussen kann. TMP-SMX kann dabei zu tatsächlichem akutem Nierenversagen (AKI) führen, aber auch durch Hemmung der Kreatininsekretion in der Niere ein AKI vortäuschen. Der vorliegende Fall zeigt eine erhebliche, bislang in diesem Ausmaß noch nicht berichtete Erhöhung von Kreatinin- und Harnstoffwerten und eine reduzierte glomeruläre Filtrationsrate bei unveränderten Cystatin-C-Werten.

## Abstract (en)

Cotrimoxazole is a combination antibiotic composed of the two active substances trimethoprim and sulfamethoxazole (TMP-SMX). TMP-SMX is known to affect renal function parameters. It can cause true acute kidney injury but may also mimic acute kidney injury by inhibiting the renal secretion of creatinine. The present case demonstrates a significant increase in creatinine and urea levels, along with a reduced glomerular filtration rate, while cystatin C levels remained unchanged, representing an extent of alteration not previously reported in this form.

## Anamnese

Einem 76-jährigen männlichen Patienten wurde aufgrund von Bauchschmerzen durch den Hausarzt eine antibiotische Therapie mit TMP-SMX in oraler Standarddosierung (960 mg zweimal pro Tag) verschrieben. Vier Tage später stellte sich der Patient aufgrund von persistierenden Beschwerden in einem auswärtigen Krankenhaus vor. Dort zeigten sich mit 15,2 mg/dl deutlich erhöhte Serumkreatininwerte. Eine vorbestehende Nierenfunktionseinschränkung war nicht bekannt. Aufgrund dieser Werte wurde die Diagnose eines AKI gestellt. Daneben zeigten sich deutlich erhöhte Entzündungswerte mit einem CRP von 14 mg/dl. Das Kalium war mit 5,5 mmol/l ebenfalls erhöht. Ein postrenales Nierenversagen konnte sonographisch ausgeschlossen werden. Zur leichteren Bilanzierung wurde ein Blasenkatheter gelegt. Dabei kam es durch eine Verletzung der Prostata zu einer Makrohämaturie. Noch am selben Tag erfolgte eine Verlegung in unsere Notaufnahme.

## Befunde

Der Blutdruck lag bei 143/89 mm Hg, die Herzfrequenz bei 82/min. Die Serumkreatininwerte lagen bei Übernahme bei 10,8 mg/dl (955 μmol/l; Normwert 0,7 bis 1,2 mg/dl; 62 bis 106 μmol/l), die Harnstoffwerte waren mit 215 mg/dl (Normwert 17 bis 49 mg/dl) ebenfalls deutlich erhöht. Die Cystatin-C-Werte waren mit 1,13 mg/l (Normwerte 0,6 bis 1,10 mg/l) lediglich grenzwertig erhöht. Die auf der Basis der Kreatininwerte errechnete glomeruläre Filtrationsrate (eGFR; CKD-EPI) lag unter 10 ml/min pro 1,73 m^2^ (Normwert ≥ 60 ml/min pro 1,73 m^2^), die eGFR berechnet auf der Grundlage der Cystatin-C-Werte lag bei 62 ml/min pro 1,73 m^2^. In der Urinanalyse zeigte sich eine Leukozyturie. Die Erythrozyturie sowie die Eiweißdiagnostik im Urin waren aufgrund der Makrohämaturie diagnostisch nicht verwertbar. Weitere auffällige Laborbefunde waren ein mit 5,3 mmol/l (Normwert 3,5 bis 5,1 mmol/l) erhöhtes Kalium, ein CRP von 15 mg/dl (Normwert ≤ 0,5 mg/dl), ein Interleukin‑6 von 36 pg/ml (Normwert ≤ 7 pg/ml), eine LDH von 355 U/l (Normwert ≤ 249 U/l) und ein Parathormon (iPTH) von 112 pg/ml (Normwert 15 bis 65 pg/ml). In der Blutgasanalyse zeigte sich eine metabolische Azidose mit einem pH-Wert von 7,288, einem Bikarbonat von 14,6 mmol/l und einem Base Excess von −12 mmol/l. Das Laktat lag bei 1,2 mmol/l. Sämtliche zusätzlich durchgeführten immunserologischen, mikrobiologischen und bildgebenden Untersuchungen erbrachten keine pathologischen Ergebnisse.

## Klinischer Verlauf und Verlauf der Laborbefunde

**Abb. 1:**
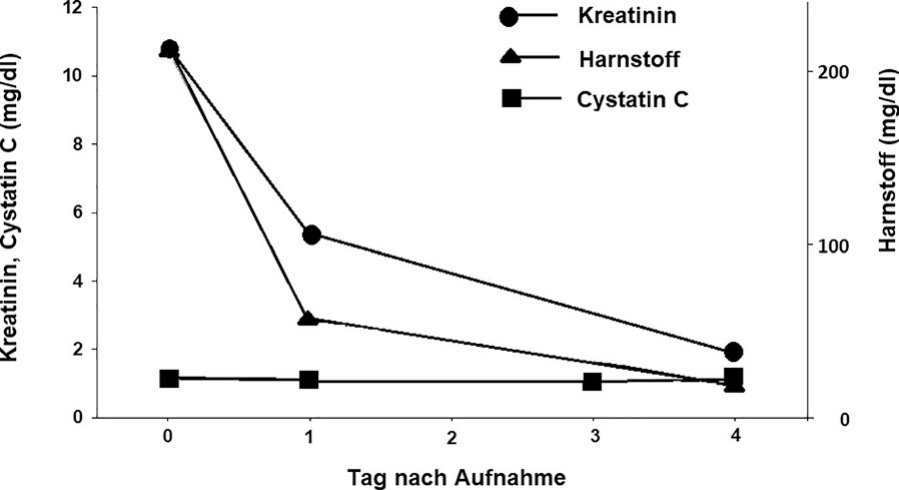
Verlauf der Kreatinin‑, Harnstoff- und Cystatin-C-Werte während der stationären Behandlung

**Abb. 2:**
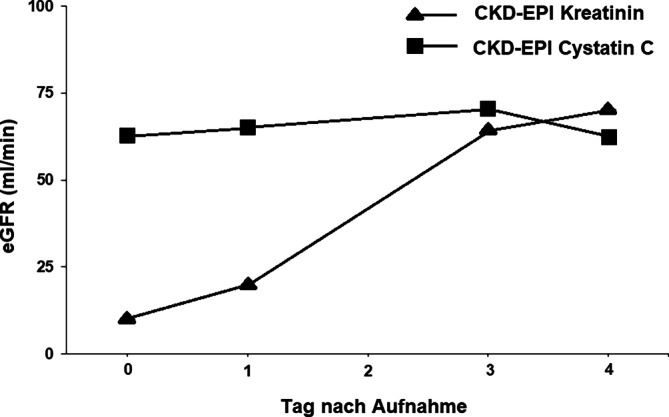
Verlauf der eGFR (CKD-EPI) basierend auf den Kreatinin- und Cystatin-C-Werten während der stationären Behandlung

Die Kreatininwerte fielen bereits am Folgetag nach Übernahme auf 2,9 mg/dl (256 μmol/l) ab und lagen am 4. Tag nach Übernahme bei 1,0 mg/dl (88 μmol/l). Auch die Harnstoffwerte lagen bei Entlassung wieder im Normbereich (37 mg/dl). Die Cystatin-C-Werte blieben konstant im oberen Normbereich mit 1,09 mg/l am Folgetag nach Aufnahme und bei 1,13 mg/l am 4. Tag nach Aufnahme (Abb. 1). Die eGFR berechnet auf Basis der Kreatininwerte besserte sich rasch und lag bei Entlassung bei 73 ml/min pro 1,73 m^2^, die eGFR berechnet auf Grundlage der Cystatin-C-Werte blieb im Verlauf konstant und lag bei Entlassung bei 62 ml/min pro 1,73 m^2^ (Abb. 2). Aufgrund der Beschwerden und der Leukozyturie wurde a. e. von einem Harnwegsinfekt als Infektquelle ausgegangen. Von einer Fortführung oder einem Wechsel der antibiotischen Therapie wurde abgesehen. Im Verlauf kam es sowohl zu einer klinischen Besserung als auch zu einem deutlichen Rückgang der Entzündungsparameter. Unter der intravenösen Gabe von 100 ml Natriumhydrogenkarbonat 8,4 % kam es zu einem ausgeglichen Säure-Basen-Status und einer Normalisierung der Kaliumwerte.

## Diskussion

TMP-SMX ist ein effektives Therapeutikum bei Weichgewebs- und Harnwegsinfektionen [8]. Seit Langem ist bekannt, dass eine Therapie mit TMP-SMX zu AKI führen kann. Histologisch zeigt sich bei diesen Patienten in der Nierenbiospie eine interstitielle Nephritis. Für das Entstehen der interstitiellen Nephritis ist der Wirkstoff Sulfamethoxazol des Kombinationsantibiotikums verantwortlich [5]. Ergänzend zu früheren Fallserien existieren mittlerweile zahlreiche systemische, meist retrospektive Analysen, die die Inzidenz und die Schwere des AKI nach TMP-SMX untersuchen [8, 14]. Die Inzidenz eines AKI wird in einer retrospektiven Analyse einer Population von 573 Patienten mittleren Alters mit 11 % angegeben. AKI wurde hier durch den Anstieg der Kreatinin- und Harnstoffwerte im Serum definiert [8]. In einer anderen großen Studie an über 2 Mio. Patienten wurde die Inzidenz des AKI nach TMP-SMX-Therapie mit etwa 14 % ähnlich hoch angegeben [13]. Bei Kindern wird mit 21–24 % sogar eine noch höhere Inzidenz berichtet [16]. Serumkreatinin wird durch verschiedene Faktoren wie Alter, Geschlecht, Muskelmasse und Ernährung beeinflusst. Kreatinin wird glomerulär filtriert und tubulär durch verschieden Transporter sekretiert [12]. TMP-SMX ist in der Lage, bei gesunden Probanden die tubuläre Kreatininsekretion zu hemmen. Dies führt zu einem Anstieg der Serumkreatininwerte, ohne dass die tatsächliche Nierenfunktion dadurch beeinflusst wird. Die verminderte Kreatininsekretion wird durch den Wirkstoff Trimethoprim vermittelt. Dieser Effekt kann bis zu einigen Tagen anhalten [15]. Der Anstieg der Kreatininwerte im Serum lag zwischen 13 und 23 %, wobei die GFR um etwa 26 % abnahm. Die durch die ^51^Cr-EDTA-Clearance ermittelte GFR zeigte dagegen keine signifikanten Veränderungen [6, 10]. Der Anstieg der Kreatininwerte im Serum war bei Patienten mit vorbestehender Nierenfunktionseinschränkung sogar noch deutlicher und lag bei bis zu 35 % [3, 11]. Clearance-Messungen mithilfe von Radioisotopen oder Inulin als traditionelle Goldstandardverfahren sind sehr aufwendig und ihr Einsatz ist in der klinischen Routinediagnostik nicht praktikabel.

Cystatin C wird glomerulär frei filtriert und dann in den Tubuluszellen absorbiert, wo es vollständig abgebaut wird [9]. TMP-SMX sollte somit keinen Effekt auf die Cystatin-C-Werte im Serum haben [6].

Eine erhöhte Inzidenz von Hyperkaliämien unter einer TMP-SMX-Therapie ist bereits vorbeschrieben. Vor allem bei älteren Patienten, die noch zusätzlich ACE-Hemmer oder Angiotensinrezeptorblocker einnehmen, war das Risiko einer Hyperkaliämie bis zu 7fach erhöht im Vergleich zu einer Therapie mit einem anderen Antibiotikum [1, 4]. Während der Grad der Nierenfunktionseinschränkung unter der Einnahme von TMP-SMX häufig überschätzt wird bzw. eine echte Nierenfunktionseinschränkung gar nicht besteht, handelt es sich bei der Hyperkaliämie um eine reale Komplikation. Hierfür gibt es verschiedene Gründe.

Trimethoprim zeigt strukturelle und pharmakologische Ähnlichkeiten zum kaliumsparenden Diuretikum Amilorid und reduziert die renale Kaliumausscheidung um etwa 40 % [7, 17]. Daneben bewirkt es eine Kaliumretention durch direkte Inhibierung des tubulären Transports über den epithelialen Na^+^-Kanal (ENaC). Trimethoprim reduziert auch die renale H^+^-Sekretion, was, wie auch in unserem Fall beobachtet, zu einer metabolischen Azidose führen kann [2, 17].

Nur wenige Studien diskutieren ihre Ergebnisse zur Inzidenz eines AKI unter TMP-SMX kritisch, in Kenntnis des möglichen Effekts von TMP-SMX auf die tubuläre Sekretion von Kreatinin und somit einer nur vorgetäuschten Einschränkung der Nierenfunktion.

Das Besondere an unserem Fall liegt zum einen darin, dass Serumkreatinin- und Harnstoffwerte bereits sehr früh im klinischen Verlauf gemessen wurden. Die Werte waren zu diesem Zeitpunkt inadäquat hoch und lagen deutlich über den berichteten Veränderungen dieser Werte nach TMP-SMX-Therapie. Der sehr rasche Abfall der Kreatinin- und Harnstoffwerte passt ebenfalls nicht zum klinischen Verlauf eines intrarenalen Nierenversagens in Form einer interstitiellen Nephritis, wie man sie als typische Komplikation bei einer TMP-SMX-Therapie erwarten würde. Hinweise auf eine prä- oder postrenale Ursache des Nierenversagens ergaben sich nicht. Die Immunserologie erbrachte nur negative Befunde, die Urindiagnostik war aufgrund der Makrohämaturie nicht verwertbar. Hätte man die Werte erst einen Tag später bestimmt, wären die Veränderungen in den Kreatinin- und Harnstoffwerten bereits weit geringer gewesen und man hätte die Veränderungen möglicherweise als klassisch im Sinne eines AKI nach TMP-SMX interpretiert, ohne weitere Überlegungen anzustellen.

Zudem wurde bereits am ersten Tag zusätzlich eine Bestimmung der Cystatin-C-Werte durchgeführt, die in der Folge mehrfach wiederholt wurde. Durch den Vergleich der Cystatin-C-Werte, die sich konstant im oberen Bereich bewegten, und der anfangs deutlich erhöhten Kreatinin- und Harnstoffwerte konnte gezeigt werden, dass es sich nicht um ein tatsächliches Nierenversagen handelte, sondern nur um eine TMP-SMX-induzierte Erhöhung bestimmter Laborparameter. Die Beurteilung der eGFR muss in solchen Situationen mit Einschränkungen erfolgen, da die gängigen Formeln zur Berechnung der eGFR stabile Serumkreatininwerte voraussetzen. Dies ist bei chronischen Prozessen der Fall, nicht aber bei akuten Veränderungen, wie sie beim akuten Nierenversagen auftreten.

Obwohl sich die Cystatin-C-Werte während der gesamten Beobachtungszeit im oberen Normbereich befanden und sowohl die Kreatinin- als auch die Harnstoffwerte bei Entlassung wieder normal waren, könnten die erhöhten Parathormonwerte ohne Hinweis auf einen primären Hyperparathyreoidismus doch für eine chronisch leicht eingeschränkte Nierenfunktion sprechen. Dies könnte zusätzlich erklären, warum der TMP-SMX-Effekt auf die renale Kreatinin- und Harnstoffsekretion in unserem Fall so ausgeprägt war im Vergleich zum Effekt bei gesunden Individuen mit normaler Nierenfunktion.

Unserer Kenntnis nach wurden solche ausgeprägten Differenzen zwischen Kreatinin- und Harnstoffwerten auf der einen Seite und Cystatin-C-Werten auf der anderen Seite bislang noch nicht publiziert.

## Fazit für die Praxis

Die Diagnose eines akuten Nierenversagens nach TMP-SMX-Therapie sollte stets kritisch hinterfragt werden. Veränderungen in Kreatinin- und Harnstoffwerten können durch hemmende Effekte von TMP-SMX auf die renale Sekretion dieser Substanzen bedingt sein, ohne dass eine tatsächliche Einschränkung der Nierenfunktion vorliegt. Hilfreich kann hier die gleichzeitige Bestimmung von Laborparametern wie Cystatin C sein, deren Serumkonzentrationen nicht durch TMP-SMX beeinflusst werden.
